# Impacto dos Índices Aterogênicos em Estenose do Enxerto de Veia Safena

**DOI:** 10.36660/abc.20190683

**Published:** 2020-09-18

**Authors:** Fethi Yavuz, Salih Kilic, Mehmet Kaplan, Arafat Yıldırım, Mehmet Kucukosmanoglu, Mustafa Dogdus

**Affiliations:** 1 Health Sciences University Adana Research and Training Hospital Department of Cardiology Adana Turquia Health Sciences University, Adana Research and Training Hospital, Department of Cardiology, Adana - Turquia; 2 Uşak University Research and Training Hospital Department of Cardiology Uşak Turquia Uşak University Research and Training Hospital, Department of Cardiology, Uşak – Turquia

**Keywords:** Veia Safena/transplante, Veia Safena/estenose, Aterosclerose, Placa Aterosclerótica, LDL Colesterol, HDL Colesterol, Hipertensão, Diabetes Mellitus, Acidente Vascular Cerebral

## Abstract

**Fundamento:**

Os enxertos de veias safenas (EVS) são frequentemente usados em pacientes submetidos a cirurgia de revascularização do miocárdio (CRM).

**Objetivos:**

Avaliar as relações entre índices aterogênicos e estenose de EVS. Métodos: No total, 534 pacientes (27,7% mulheres, com idade média de 65±8,4 anos) submetidos a CRM e angiografia coronariana eletiva foram incluídos no estudo. Pacientes com pelo menos uma estenose EVS ≥50% foram alocados ao grupo estenose EVS (+) (n=259) e pacientes sem estenose foram classificados como EVS (-) (n=275). O índice aterogênico plasmático (IAP) e o coeficiente aterogênico (CA) foram calculados a partir dos parâmetros lipídicos de rotina dos pacientes. A significância foi estabelecida no nível p<0,05.

**Resultados:**

O número de pacientes com histórico de hipertensão (HT), diabetes mellitus (DM), acidente vascular cerebral e insuficiência cardíaca (IC) se mostrou significativamente maior no grupo EVS (+) do que no grupo EVS (-). O colesterol total, triglicerídeos e colesterol LDL mostraram-se significativamente mais altos e o colesterol HDL mostrou-se menor no grupo EVS (+) do que no grupo EVS (-). IAP (p<0,001) e CA (p<0,001) apresentaram-se significativamente mais altos no grupo EVS (+) do que no grupo EVS (-). A análise ROC mostra que tanto o IAP quanto o CA mostraram-se melhores que o colesterol HDL, colesterol LDL e colesterol não HDL na predição de estenose de EVS. Na análise multivariada, histórico de DM, HT, acidente vascular cerebral, IC, número de enxertos de safena, colesterol HDL, colesterol LDL, colesterol não HDL, IAP e CA foram fatores de risco independentes para estenose de EVS.

**Conclusão:**

O IAP e o CA foram preditores independentes de estenose de EVS. Além disso, tanto o IAP quanto o CA têm melhor desempenho na predição de estenose de EVS do que o colesterol LDL, colesterol HDL e colesterol não HDL. (Arq Bras Cardiol. 2020; 115(3):538-544)

## Introdução

Embora a cirurgia de revascularização do miocárdio (CRM) tenda a usar enxertos arteriais, os enxertos de veias safenas (EVS) ainda são frequentemente utilizados, principalmente em situações de emergência e em lesões de múltiplos vasos.^1^ O EVS tende a degenerar com o tempo. A deficiência de EVS limita o sucesso a longo prazo da cirurgia de revascularização miocárdica. As taxas de patência no 1º, 5º e no 10º ano após a CRM foram 93%, 74% e 41%, respectivamente.^2^

As principais causas de deficiência de EVS são trombose no período inicial (<1 mês), hiperplasia neointimal no período subagudo (1–12 meses) e aterosclerose no período tardio.^3-5^

Sabe-se que os níveis lipídicos séricos estão fortemente associados à aterosclerose.^6^ Níveis elevados de colesterol de lipoproteína de alta densidade (colesterol HDL) proporcionam um efeito cardioprotetor, enquanto níveis elevados de colesterol de lipoproteína de baixa densidade (colesterol LDL) são aterogênicos. Além disso, muitos estudos demonstraram que altos níveis de triglicerídeos (TG) estão associados à doença arterial coronariana (DAC).^7-9^ No entanto, a relação entre aumento dos parâmetros lipídicos e aumento do risco de doença arterial coronariana (DAC) permanece controversa. Estudos anteriores que mostram a associação entre colesterol e aterosclerose foram realizados usando os níveis de colesterol total (CT) e LDL-C. Quando do surgimento do papel do colesterol HDL como agente cardioprotetor, o uso da razão colesterol LDL/colesterol HDL foi recomendado para determinar o risco de DAC. Como os efeitos dos níveis séricos elevados de TG na aterosclerose estão claramente definidos, essa razão tem valor limitado, pois não inclui os níveis de TG.^10^

É importante ressaltar que diversas razões de lipoproteínas ou índices aterogênicos podem ser usados para otimizar a capacidade preditiva do perfil lipídico. Estudos demonstraram que os índices lipídicos calculados a partir dos parâmetros do perfil lipídico apresentam melhor valor preditivo em doenças cardiovasculares.^11^ O colesterol não HDL é recomendado como um preditor mais provável de DAC do que o colesterol LDL, porque representa o teor de colesterol encontrado em todas as lipoproteínas aterogênicas.^12^ O índice aterogênico plasmático (IAP) é calculado por meio de dois parâmetros importantes: TG sérico e colesterol HDL sérico. O uso simultâneo de triglicerídeos e colesterol HDL para essa razão reflete múltiplas interações entre o metabolismo de diferentes lipoproteínas e pode ser útil para predizer a aterogenicidade plasmática.^13^ Outro índice é o coeficiente aterogênico (CA) calculado como a razão entre colesterol não HDL e HDL.^14^ Até onde sabemos, a associação de índices aterogênicos plasmáticos com doença de EVS não foi estudada. Neste estudo, investigamos a relação entre os parâmetros lipídicos e os índices aterogênicos IAP e CA na estenose de EVS.

### Materiais e Métodos

O estudo incluiu 534 pacientes (27,7% mulheres, n=148) submetidos a angiografia coronariana eletiva por mais de um ano (média de 5,3 anos) após a cirurgia de revascularização miocárdica, que envolveu pelo menos um EVS para *bypass*. Os pacientes foram divididos em dois grupos de acordo com a extensão da patência do EVS. Estenose de 50% ou mais, dentro do EVS, foi categorizada como estenose de EVS (+).

O comitê de ética local aprovou o protocolo deste estudo, conduzido de acordo com os princípios estabelecidos na Declaração de Helsinque (22/05/2019).

As características clínicas e demográficas basais da população do estudo foram registradas no formulário preparado para cada paciente. Todos os parâmetros laboratoriais de rotina antes da angiografia coronariana, laudo ecocardiográfico, peso, altura e índice de massa corporal (IMC) foram registrados no sistema digital do hospital. Calculou-se o IMC como peso/altura (m^2^). A hipertensão foi definida como medidas sistêmicas repetidas da pressão arterial que excederam 140/90 mmHg ou se o paciente estivesse tomando medicação anti-hipertensiva. Diagnosticou-se diabetes mellitus (DM) de acordo com um dos seguintes critérios: (1) glicemia em jejum ≥126 mg/dL, (2) glicemia >200 mg/dL a qualquer momento, (3) histórico de DM ou pacientes sob medicação antidiabética. Definiu-se hipercolesterolemia como nível basal total de colesterol >200 mg/dL ou tratamento atual com estatinas e/ou agentes hipolipemiantes. Fumantes atuais foram os que relataram tabagismo regular nos 6 meses anteriores. Definiu-se histórico familiar de doença arterial coronariana como a presença de doença arterial coronariana em parente de primeiro grau masculino com idade <55 anos ou em parente de primeiro grau feminino com idade <65 anos.

### Cálculo de Índices Aterogênicos

O colesterol não HDL foi calculado como a diferença entre colesterol total e colesterol de alta densidade (colesterol total sérico — HDL-C sérico).^15^

O IAP foi calculado como o logaritmo da razão sérica de triglicerídeos/colesterol HDL sérico.^13,16^

O coeficiente aterogênico (CA) foi calculado como a razão entre colesterol não HDL e HDL, da seguinte forma: (colesterol sérico total — HDL-C sérico)/colesterol HDL).^14^

### Avaliação Angiográfica

A angiografia coronariana foi realizada rotineiramente pela técnica de Judkins, com 6 ou 7 cateteres cardíacos franceses direito e esquerdo. Os angiogramas foram gravados em mídia digital DICOM a 25 quadrados/ms e foram revisados por dois angiógrafos experientes que desconheciam o estado clínico dos pacientes. Os enxertos de veias safenas foram vistos a partir de pelo menos dois ângulos após injeção seletiva de material de contraste. Definiu-se doença de enxerto venoso como estenose de 50% do diâmetro do vaso em qualquer EVS.

### Análise Estatística

Variáveis contínuas com distribuição normal foram descritas como média ± desvio padrão (DP) e variáveis sem distribuição normal foram descritas como mediana e intervalo interquartil. As variáveis categóricas são apresentadas como o número de pacientes e porcentagens. As variáveis foram investigadas pelo método analítico de Kolmogorov-Smirnov para determinar se apresentam distribuição normal ou não. Realizou-se teste *t* de Student não pareado para variáveis com distribuição normal e o teste U de Mann-Whitney para aquelas sem distribuição normal. Para as variáveis categóricas, utilizou-se o teste do qui-quadrado ou o exato de Fisher. O tamanho da amostra da população do estudo não foi calculado e todos os pacientes consecutivos em nossa clínica foram incluídos no estudo.

Foram utilizadas curvas *receiver operating characteristic* (ROC) para demonstrar a sensibilidade e especificidade dos índices e lipídios, e seus valores de corte para prever estenose de EVS. A área sob a curva (AUC) desses índices aterogênicos e lipídios foi calculada pelo método DeLong.^17^ Para calcular as *hazard ratios* (HR) e seus intervalos de confiança de 95% (IC 95%) para estenose de EVS, realizou-se análise de regressão logística univariada e multivariável. Valores de p<0,05 foram considerados estatisticamente significativos. Os dados foram analisados utilizando o software estatístico SPSS, versão 20.0 (SPSS Inc., Chicago, IL, EUA) e o software estatístico MedCalc 15 (Ostend, Bélgica).

## Resultados

No total, 534 pacientes (idade média de 65±8,4 anos) foram incluídos no estudo. Desse número, 259 (48,5%) foram diagnosticados com estenose de EVS (EVS (+)) e 275 (51,5%) foram diagnosticados com patência de EVS (EVS (-)). As características demográficas e clínicas basais dos grupos estudados estão resumidas na Tabela 1. O número de pacientes com histórico de HT, DM, acidente vascular cerebral e insuficiência cardíaca se mostrou significativamente maior no grupo estenose EVS (+) do que no grupo estenose EVS (-). A pressão arterial sistólica média e o número de enxertos foram maiores no grupo estenose EVS (+) do que no grupo estenose EVS (-). A fração de ejeção ventricular esquerda foi menor no grupo estenose EVS (+) do que no grupo estenose SVG (-). Os parâmetros laboratoriais e os índices lipídicos dos grupos de pacientes são apresentados na Tabela 2. Os parâmetros lipídicos, i.e., colesterol total, triglicerídeos e colesterol LDL apresentaram-se significativamente maiores, e o colesterol HDL apresentou-se menor no grupo EVS (+) do que no grupo EVS (-). Os índices lipídicos, IAP, CA e colesterol não HDL mostraram-se significativamente mais altos no grupo estenose EVS (+) do que no grupo estenose EVS (-) (p<0,001, para todos).

**Tabela 1:** – Comparação das características basais clínicas da população estudada

Variável	Estenose de EVS (+) (n=259)	Estenose de EVS (-) (n=275)	p
Idade, anos	65,9 ± 8,66	64,6 ± 8,22	0,071
Índice de massa corporal, kg/m^2^	28,1 ± 3,75	28,0 ± 4,70	0,709
Pressão arterial sistólica, mmHg	126,5 ± 17,55	123,0 ± 12,24	0,007
Pressão arterial diastólica, mmHg	75,2 ± 9,89	74,0 ± 8,38	0,141
Intervalo após a cirurgia, mês	67,5 ± 39,55	60,6 ± 29,53	0,171
Fração de ejeção ventricular esquerda, %	52,1 ± 9,48	55,8 ± 6,38	<0,001
Sexo feminino, % (n)	25,1 (65)	30,4 (83)	0,172
Hipertensão, % (n)	78,0 (202)	66,5 (183)	0,003
Diabetes mellitus, % (n)	62,5 (162)	46,5 (128)	<0,001
Hiperlipidemia, % (n)	77,6 (201)	76,2 (204)	0,356
Doença vascular periférica, % (n)	12,7 (33)	9,5 (26)	0,226
Acidente vascular cerebral, % (n)	17,8 (46)	8,7 (24)	0,002
Doença renal crônica, % (n)	3,9 (10)	2,9 (8)	0,542
Insuficiência cardíaca, % (n)	38,6 (100)	15,6 (43)	<0,001
Fibrilação atrial, % (n)	6,2 (16)	6,6 (18)	0,827
Número de enxertos	1,99 ± 0,7	1,7 ± 0,65	<0,001
Aspirina, % (n)	81,1 (210)	85,8 (236)	0,140
Inibidores de P2Y12, % (n)	32,4 (84)	29,5 (81)	0,457
Betabloqueadores, % (n)	87,6 (227)	85,1 (234)	0,391
IECA/BRA, % (n)	75,7 (196)	71,6 (197)	0,290
Estatina, % (n)	59,8 (155)	64,7 (178)	0,245
Diuréticos, % (n)	22,8 (59)	15,3 (42)	0,027
Trimetazidina, % (n)	24,3 (63)	27,3 (75)	0,437
Ranolazina, % (n)	17,8 (46)	18,9 (52)	0,732
Nitratos, % (n)	27,8 (72)	16,7 (46)	0,002
Bloqueadores de canais de cálcio, % (n)	18,9 (49)	19,6 (54)	0,834

*IECA: Inibidor da enzima conversora da angiotensina; BRA: bloqueador do receptor de angiotensina.*

**Tabela 2:** – Comparação de parâmetros laboratoriais e índices lipídicos dos grupos de estudo

Variável	Estenose de EVS (+) (n=259)	Estenose de EVS (-) (n=275)	p
Hba1c, %	7,2 ± 1,9	7,0 ± 2,0	0,296
Glicemia de jejum, md/dL	174 ± 108	164 ± 94	0,249
Hemoglobina, g/dL	13,2 ± 1,64	13,1 ± 1,84	0,205
Hematócrito, %	39,1 ± 4,82	38,6 ± 4,97	0,259
Contagem de glóbulos brancos, x10^3^/mL	8,7 ± 2,26	8,5 ± 2,1	0,553
Contagem de plaquetas, x10^3^/mL	244 ± 68	237 ± 65	0,206
Contagem de neutrófilos, x10^3^/mL	5,5 ± 1,78	5,3 ± 1,93	0,307
Contagem de linfócitos, x10^3^/mL	2,1 ± 0,8	2,1 ± 0,7	0,377
Contagem de monócitos, x10^3^/mL	0,7 ± 0,21	0,7 ± 0,26	0,860
Volume médio plaquetário, fL	8,8 ± 0,96	9,9 ± 1,12	0,123
*Red cell distribution width*, fL	15,7 ± 2,71	14,7 ± 2,14	0,182
Colesterol total, mg/dL, mediano (percentil 25^o^–75^o^)	201 (174–232)	185,0 (165–222)	0,001
Colesterol LDL, mg/dL	137,0 ± 34,7	126,8 ± 37,2	0,001
Colesterol HDL, mg/dL, mediano (percentil 25^o^–75^o^)	37,0 (34,0–43,0)	48,0 (42–54,5)	<0,001
Triglicerídeos, mg/dL, mediano (percentil 25^o^–75^o^)	203 (156–273)	143,0 (111–189,5)	<0,001
Ureia, mg/dL, mediana (percentil 25^o^–75^o^)	37,0 (29,0–47,0)	37,0 (29,0–42,0)	0,621
Creatinina, mg/dL, mediana (percentil 25^o^–75^o^)	0,90 (0,70.–1.10)	0,80 (0,70–1,0)	0,253
Albumina, mg/dL	3,8 ± 0,49	3,9 ± 0,38	0,174
Alanina transaminase, U/L	19,1 ± 2,93	19,0 ± 3,17	0,899
Aspartato aminotransferase, U/L	23,2 ± 3,61	24,2 ± 3,94	0,966
Ácido úrico sérico, mg/dL	5,7 ± 1,6	5,4 ± 1,7	0,172
IAP	0,73 ± 0,20	0,50 ± 0,23	<0,001
CA	4,44 ± 1,30	3,08 ± 1,06	<0,001
Colesterol não HDL	165,7 ± 42,74	144,6 ± 44,44	<0,001

*CA: Coeficiente aterogênico; IAP: Índice aterogênico plasmático; HDL: Lipoproteína de alta densidade, LDL: Lipoproteína de baixa densidade.*

Para a predição de estenose de EVS, o valor de corte de 3,4<CA possui sensibilidade de 79,15% e especificidade de 66.18%; o valor de corte de 0.56<IAP possui 83,01% de sensibilidade e 67,53% de especificidade; colesterol HDL<40 mg/dL possui 64,9% de sensibilidade e 84,0% de especificidade; 121 mg/dL<colesterol LDL possui 78,4% de sensibilidade e 58,9% de especificidade; e 141<colesterol não HDL possui 70,66% de sensibilidade e 54,55% de especificidade nas análises da curva ROC (Figura). Comparações pareadas da análise ROC mostram que, embora não houvesse diferenças significativas entre o IAP e o CA, tanto o IAP quanto o CA mostraram-se superiores ao colesterol HDL, colesterol LDL e colesterol não HDL na predição de estenose de EVAS.

**Figura 1:**
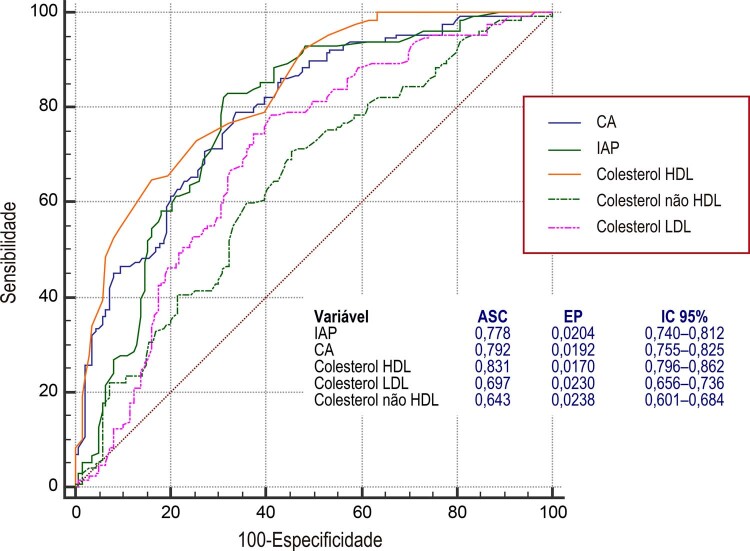
– Curvas receiver operating characteristic (ROC) para estenose de EVS.

Para determinar os fatores de risco independentes para estenose de EVS, realizou-se análise logística univariada e multivariada (Tabela 3). Na análise multivariada, histórico de DM, HT, acidente vascular cerebral, IC, número de enxertos de safena, colesterol HDL, colesterol LDL e colesterol não HDL foram fatores de risco independentes para estenose de EVS. Além disso, os índices aterogênicos IAP e CA foram preditores independentes de estenose de EVS. Embora o nível de triglicerídeos tenha sido significativo na análise univariada, ele não alcançou significância estatística na análise multivariada.

## Discussão

No presente estudo, observamos que fatores de risco cardiovascular tradicionais, como HT, DM, acidente vascular cerebral e insuficiência cardíaca, foram preditores independentes de estenose de EVS. Além disso, descobrimos que o colesterol HDL, colesterol LCL, colesterol não HDL, número de EVS e os índices aterogênicos IAP e CA foram preditores independentes de estenose de EVS. Além disso, observamos que o IAP e o CA têm melhor desempenho na predição de estenose de EVS do que o colesterol LDL, colesterol HDL e colesterol não HDL.

A estenose do enxerto de veia safena é uma questão importante no curto e no longo prazo dos pacientes submetidos a CRM, estando relacionada a grandes eventos cardiovasculares adversos. É importante identificar pacientes com alto risco de estenose de EVS para estratégias de prevenção e tratamento. Muitos fatores de risco relacionados à estenose de EVS, como fatores cirúrgicos, tabagismo, HT, HPL, DM, acidente vascular cerebral e outros, já foram determinados. Em seus estudos, Deppe et al. e Kim et al. mostraram que fatores cirúrgicos, como técnicas de aquisição de vasos ou uso de circulação extracorpórea, foram preditores independentes de estenose de EVS.^18,19^

O tabagismo é um fator de risco bem conhecido para aterosclerose e doença arterial coronariana.^20-22^ Sen et al. demonstraram que o tabagismo é um fator de risco independente para a doença de EVS em sua análise de regressão multivariada.^20-22^ Semelhante a estudos anteriores, demonstramos que o tabagismo é um importante fator de risco para estenose de EVS.

Demonstrou-se que a DM é uma das principais causas de aterosclerose em diversos estudos.^23^ A DM pode causar estenose de EVS como resultado de disfunção endotelial vascular causada por mecanismos fisiopatológicos imunes e não imunes.^24^ Koshizaka et al.,^25^ examinaram a relação entre DM e falha de enxerto avaliada por angiografia de um ano e desfecho clínico de cinco anos em pacientes submetidos a CRM. No estudo, observou-se que que a falha no EVS no primeiro ano foi semelhante entre pacientes com e sem DM. No entanto, óbito, infarto do miocárdio ou revascularização mostraram-se significativamente maiores em pacientes com DM com desfecho clínico de 5 anos.^25^ Em nosso estudo, histórico de DM foi considerado fator de risco independente para estenose de EVS.

A dislipidemia é um fator de risco bem conhecido para aterosclerose.^23^ Há evidências crescentes de que colesterol HDL baixo e colesterol LDL alto, colesterol total e triglicerídeos desempenham um papel na progressão da aterosclerose e doença arterial coronariana.^26^ Da mesma forma, demonstrou-se que colesterol HDL baixo com colesterol LDL alto está associado à estenose de EVS.^27,28^ Entre esses parâmetros, recomenda-se que o nível de colesterol LDL seja selecionado como alvo do tratamento.^26^ No entanto, após reduzir o colesterol LDL para os níveis recomendados, permanece um risco cardiovascular de ∼50%, motivando os pesquisadores a encontrarem novos preditores de arteriosclerose.^29^ Em comparação com os parâmetros lipídicos únicos, os índices lipídicos abrangentes, como IAP, CA e não HDL, são considerados melhores preditores de aterosclerose e doenças arteriais coronarianas.^30,31^

Em nosso estudo, semelhante a estudos anteriores, baixos níveis de colesterol HDL e altos níveis de colesterol LDL foram considerados fatores de risco independentes para estenose de EVS. Além disso, o alto nível de colesterol não HDL, outro fator de risco aterogênico, foi considerado um preditor independente de estenose de EVS.O colesterol não HDL não é o primeiro alvo na terapia hipolipemiante e pode ser um alvo secundário quando os pacientes atingem os níveis recomendados de colesterol LDL.^26^ Em nosso estudo, o nível médio de colesterol LDL dos pacientes foi superior aos valores recomendados para prevenção secundária. Isso mostra que os pacientes não receberam o tratamento hipolipemiante necessário e aqueles que o receberam não usaram doses efetivas para o colesterol LDL, que é o alvo principal.

Como se sabe, a LDL pequena e densa (sdLDL) pode se acumular e oxidar mais facilmente na parede do vaso em comparação com o colesterol LDL. Estudos anteriores mostraram que a sdLDL é um marcador que prediz a aterosclerose e pode ser usado clinicamente.^26^ No entanto, é complicado medir a sdLDL no sangue e seu alto custo limita seu uso clínico. O IAP obtido a partir do logaritmo TG/colesterol HDL reflete fortemente o equilíbrio entre lipoproteínas aterogênicas e protetoras. Um estudo anterior mostrou o IAP como inversamente relacionado ao diâmetro do colesterol LDL.^32^ Por esse motivo, o IAP pode ser usado como um fácil indicador na doença arterial coronariana. O IAP também pode ser rotineiramente calculado a partir de parâmetros lipídicos e usado como substituto do tamanho da partícula LDL sem ocasionar custos extras.

No presente estudo, observamos que o IAP é um indicador independente de estenose de EVS e apresentou melhor desempenho na predição de estenose de EVSA do que o colesterol LDL e o colesterol não HLD. Com base nesses resultados, mostramos que o IAP obtido por um cálculo simples pode ser usado para prever estenose de EVS. Em estudos anteriores, os valores médios do IAP diferiram.^15,33-35^ Essas diferenças podem dever-se à não homogeneidade de grupos de pacientes selecionados ou ao uso de fármacos. Outro motivo pode ser a diferença na carga de aterosclerose na população. No presente estudo, avaliamos apenas pacientes submetidos a CRM sob alto risco aterosclerótico. Como resultado, o valor médio do IAP do nosso estudo foi superior ao dos estudos publicados anteriormente.

O coeficiente aterogênico (CA) é uma medida de colesterol que utiliza frações de lipoproteínas LDL-C, VLDL-C e IDL-C em relação ao colesterol HDL.^36^

Ele reflete o potencial aterogênico de todo o espectro de frações de lipoproteínas. Como destacado acima, o colesterol não HDL é o segundo alvo da terapia após o colesterol LDL, de acordo com as diretrizes da ESC (European Society of Cardiology), principalmente em indivíduos com hipertrigliceridemia.^26^ Portanto, esse índice simples pode fornecer informações importantes para a identificação de indivíduos com risco de doença cardiovascular. Em nosso estudo, observamos que o CA médio mostrou-se significativamente maior no grupo estenose EVS (+) do que no grupo EVS (-) e foi preditor independente de estenose.

Além disso, o CA mostrou melhor desempenho na análise ROC do que o colesterol não HDL. Esse resultado mostra que o CA, que é um índice simples, deve ser considerado para prever estenose de EVS.

### Limitações

O presente estudo apresenta algumas limitações. Primeiramente, trata-se de um estudo unicêntrico retrospectivo. No entanto, o número de pacientes é alto, o que aumenta o valor do estudo. Em segundo lugar, não comparamos os resultados com indivíduos controle saudáveis, o que poderia mostrar o valor normal do IAP e CA em nossa população saudável. Em terceiro lugar, como não houve desfechos clínicos no estudo, não é possível avaliar o impacto do IAP e do CA nos desfechos clínicos. Portanto, outros estudos são necessários para avaliar o impacto desses índices em pacientes submetidos a CRM.

## Conclusão

No presente estudo, mostramos que HT, DM, acidente vascular cerebral, insuficiência cardíaca e colesterol HDL, colesterol LDL e colesterol não HDL foram preditores independentes de estenose de EVS, semelhante a estudos anteriores. Além disso, até onde sabemos, este é o primeiro estudo que mostra uma associação entre estenose de EVS e os índices aterogênicos IAP e CA. Além disso, observamos que os índices aterogênicos IAP e CA apresentam melhor desempenho na predição de estenose de EVS do que os parâmetros lipídicos padrão colesterol LDL, colesterol HDL e colesterol não HDL.
