# Supplementary material for: Impacto dos Índices Aterogênicos em Estenose do Enxerto de Veia Safena
Source: Arq Bras Cardiol. 2020 Sep 18;115(3):538–44. [Article in Portuguese] doi: 10.36660/abc.20190683 (PMC9363077; doi:10.36660/abc.20190683)
Supplement: Translation

# Translation: Impact of Atherogenic Indexes in Saphenous Vein Graft Stenosis

**Authors:** Fethi Yavuz, Salih Kilic, Mehmet Kaplan, Arafat Yıldırım, Mehmet Kucukosmanoglu, Mustafa Dogdus

**Affiliations:** 1 Turkey Health Sciences University, Adana Research and Training Hospital, Department of Cardiology, Adana - Turkey; 2 Turkey Uşak University Research and Training Hospital, Department of Cardiology, Uşak – Turkey

**Keywords:** Saphenous Vein/transplantation, Saphenous Vein/Stenosis, Plaque, Atherosclerotic, LDL, Cholesterol, HDL, Cholesterol, Hypertension, Diabetes Mellitus, Stroke

## Abstract

**Background:**

Saphenous vein grafts (SVG) are frequently used in patients that have undergone coronary artery bypass graft (CABG) surgery. Objectives: To evaluate the relationship between atherogenic indexes and SVG stenosis.

**Methods:**

Altogether, 534 patients (27.7% women, mean age 65±8.4 years) that underwent CABG and elective coronary angiography were included in the study. Patients with at least one SVG stenosis ≥50% were allocated to the stenosis group SVG (+) (n=259) and patients without stenosis were categorized as SVG (-) (n=275). Atherogenic index of plasma (AIP) and atherogenic coefficient (AC) were calculated from the patients’ routine lipid parameters. The level of significance was p<0.05.

**Results:**

The number of patients with a history of hypertension (HT), diabetes mellitus (DM), stroke, and heart failure was significantly higher in the SVG (+) group than in the SVG (-) group. Total cholesterol, triglycerides, LDL-C were significantly higher and HDL-C was lower in the SVG (+) group than in the SVG (-) group. AIP (p<0.001) and AC (p<0.001) were significantly higher in the SVG (+) group than in the SVG (-) group. The receiver operating characteristic (ROC) analysis show that both AIP and AC were better than HDL-C, LDL-C and non-HDL-C at predicting SVG stenosis. In the multivariate analysis, history of DM, HT, stroke, heart failure (HF), number of saphenous grafts, HDL-C, LDL-C, non-HDL-C, AIP and AC were found to be independent risk factors for SVG stenosis.

**Conclusion:**

AIP and AC were independent predictors of SVG stenosis. Moreover, both AIP and AC have better performance in predicting SVG stenosis than LDL-C, HDL-C and non-HDL-C. (Arq Bras Cardiol. 2020; 115(3):538-544)

## Introduction

Although coronary artery bypass graft (CABG) surgery tends to use arterial grafts, saphenous vein grafts (SVG) are still frequently used, especially in emergency situations and in multiple vessel lesions.1 SVG tends to degenerate over time. SVG deficiency limits the long-term success of surgical coronary revascularization. Patency rates at the 1st, 5th and 10th years after CABG were reported to be 93%, 74% and 41%, respectively.2

The main causes of SVG deficiency are thrombosis in the early period (<1 month), neointimal hyperplasia in the subacute period (1–12 months) and atherosclerosis in the late period.^3-5^

Serum lipid levels are known to be strongly associated with atherosclerosis.^6^ Increased levels of high-density lipoprotein cholesterol (HDL-C) provide a cardioprotective effect while elevated low-density lipoprotein cholesterol (LDL-C) levels are atherogenic. In addition, many studies have shown that high triglyceride (TG) levels are associated with coronary artery disease (CAD).^7-9^ However, the relationship between increased lipid parameters and increased risk of coronary artery disease (CAD) remains controversial. Previous studies showing the association of cholesterol with atherosclerosis were performed using total cholesterol (TC) and LDL-C levels. When the role of HDL-C as a cardio-protective agent appeared, the use of the LDL-C/HDL-C ratio was recommended to determine the risk of CAD. Since the effects of high-serum TG levels on atherosclerosis are clearly defined, this ratio has a limited value since it does not include TG levels.^10^

Importantly, various lipoprotein ratios or atherogenic indexes can be used to optimize the predictive capacity of the lipid profile. Studies have shown that lipid indexes calculated from lipid profile parameters have a better predictive value in cardiovascular diseases.^11^ Non-HDL cholesterol (NHC) is recommended as a more likely predictor of CAD than LDL-C because it represents the cholesterol content found in all atherogenic lipoproteins.^12^ Atherogenic index of plasma (AIP) is calculated with two important parameters: serum TG and serum HDL-C. The simultaneous use of triglycerides and HDL-C for this ratio reflects multiple interactions between the metabolism of different lipoproteins and may be useful for predicting plasma atherogenicity.^13^Another index is the atherogenic coefficient (AC) calculated as the ratio of non-HDL cholesterol to HDL.^14^ To our knowledge, the association of plasma atherogenic indexes with SVG disease has not been studied. In this study, we investigated the relationship between lipid parameters and the atherogenic indexes AIP and AC in SVG stenosis.

### Materials and Methods

The study consisted of 534 patients (27.7% women, n=148) who underwent elective coronary angiography more than one year (mean 5.3 years) after CABG surgery, which involved at least one SVG for bypass. The patients were divided into two groups according to the extent of SVG patency. Stenosis of 50% or greater within the SVG was categorized as SVG (+) stenosis.

The local ethics committee approved the protocol of this study, which was conducted according to the principles established in the Declaration of Helsinki (22/05/2019).

Baseline clinical and demographic characteristics of the study population were recorded in the form that was prepared for each patient. All routine laboratory parameters before coronary angiography, echocardiography report, weight, height and body mass index (BMI) were recorded from the digital system of the hospital. BMI was calculated as weight/height (m)^2^. Hypertension was defined as repeated systemic blood pressure measurements exceeding 140/90 mmHg or if the patient was taking anti-hypertensive medication. Diabetes mellitus (DM) was diagnosed according to one of the following criteria: (1) fasting blood glucose ≥126 mg/dL, (2) blood glucose >200 mg/dL at any time, (3) history of DM or patients on anti-diabetic medication. Hypercholesterolemia was defined as baseline total cholesterol level >200 mg/dL or current treatment with statins and/or lipid-lowering agents. Current smokers were those reporting regular smoking in the previous 6 months. Family history of coronary artery disease was defined as the presence of coronary artery disease in a male first-degree relative aged <55 years or in a female first-degree relative aged <65 years.

### Calculation of atherogenic indexes

Non-HDL was calculated as the difference between total cholesterol and high-density cholesterol (serum total cholesterol — serum HDL-C).^15^

AIP was calculated as the logarithm of serum triglyceride/serum HDL-C ratio.^13,16^

Atherogenic coefficient (AC) was calculated as the ratio of non-HDL cholesterol to HDL cholesterol, as follows: (total serum cholesterol — Serum HDL-C)/HDL-C).^14^

### Angiographic evaluation

Coronary angiography was routinely performed by the Judkins technique using 6 or 7 French right and left heart catheters. Angiograms were recorded on a DICOM digital media at 25 squares/msec and were reviewed by two experienced angiographers unaware of the patients’ clinical status. Saphenous vein grafts were viewed from at least two angles after selective injection of contrast material. Vein graft disease was defined as stenosis of 50% of the vessel diameter in any SVG.

### Statistical analysis

Continuous variables with normal distribution were reported as mean ± standard deviation (SD) and variables without normal distribution were reported as median and interquartile range. Categorical variables were expressed as the number of patients and percentages. The variables were investigated using the analytic method of Kolmogorov-Smirnov to determine whether or not they are normally distributed. Unpaired Student’s *t*-test was performed for normally distributed variables and Mann-Whitney U test for those without normal distribution. Chi-square test or Fisher’s exact test was performed for the categorical variables. The sample size of the study population was not calculated and all consecutive patients in our clinic were included in the study.

Receiver operating characteristics (ROC) curves were used to demonstrate the sensitivity and specificity of the indexes and lipids, and their cut-off values for predicting SVG stenosis. The area under the curve (AUC) of these atherogenic indexes and lipids was calculated using the DeLong method.^17^ To calculate hazard ratios (HR) and their 95% confidence intervals (95% CIs) for SVG stenosis, univariate and multivariate logistics regression analysis was performed. A p value<0.05 was considered statistically significant. Data were analyzed using SPSS statistical software, version 20.0 (SPSS Inc., Chicago, IL, USA) and MedCalc 15 statistical software (Ostend, Belgium). (Quais índices? Sugiro especificar)

## Results

A total of 534 patients (mean age 65±8.4 years) was included in the study. Of those, 259 (48.5%) were diagnosed with a stenotic SVG (SVG (+)) and 275 (51.5%) were diagnosed as patency SVG (SVG (-)). Baseline demographic and clinical characteristics of study groups are summarized in Table 1. The number of patients with a history of HT, DM, stroke, and heart failure were significantly higher in the SVG (+) stenosis group than in the SVG (-) stenosis group. Mean systolic blood pressure and number of grafts were higher in the SVG (+) stenosis group than in the SVG (-) stenosis group. Left ventricular ejection fraction was lower in the SVG (+) stenosis group than in the SVG (-) stenosis group. Laboratory parameters and lipid indexes of the patient groups are presented in Table 2. Lipid parameters, meaning, total cholesterol, triglycerides and LDL-C were significantly higher, and HDL-C was lower in the SVG (+) group than in the SVG (-) group. The lipid indexes, AIP, AC, and non-HDL-C were significantly higher in the SVG (+) stenosis group than in the SVG (-) stenosis group (p<0.001, for all).

**Table 1:** – Comparison of baseline characteristics of the study population

Variable	SVG stenosis (+) (n=259)	SVG stenosis (-) (n=275)	p
Age, years	65.9 ± 8.66	64.6 ± 8.22	0.071
Body mass index, kg/m^2^	28.1 ± 3.75	28.0 ± 4.70	0.709
Systolic blood pressure, mmHg	126.5 ± 17.55	123.0 ± 12.24	0.007
Diastolic blood pressure, mmHg	75.2 ± 9.89	74.0 ± 8.38	0.141
Time interval after surgery, month	67.5 ± 39.55	60.6 ± 29.53	0.171
Left ventricular ejection fraction, %	52.1 ± 9.48	55.8 ± 6.38	<0.001
Female, % (n)	25.1 (65)	30.4 (83)	0.172
Hypertension, % (n)	78.0 (202)	66.5 (183)	0.003
Diabetes mellitus, % (n)	62.5 (162)	46.5 (128)	<0.001
Hyperlipidemia, % (n)	77.6 (201)	76.2 (204)	0.356
Peripheral vascular disease, % (n)	12.7 (33)	9.5 (26)	0.226
Stroke, % (n)	17.8 (46)	8.7 (24)	0.002
Chronic kidney disease, % (n)	3.9 (10)	2.9 (8)	0.542
Heart failure, % (n)	38.6 (100)	15.6 (43)	<0.001
Atrial fibrillation, % (n)	6.2 (16)	6.6 (18)	0.827
Number of grafts	1.99 ± 0.7	1.7 ± 0.65	<0.001
Aspirin, % (n)	81.1 (210)	85.8 (236)	0.140
P2Y12 inhibitors, % (n)	32.4 (84)	29.5 (81)	0.457
Beta-blockers, % (n)	87.6 (227)	85.1 (234)	0.391
ACEI/ARB, % (n)	75.7 (196)	71.6 (197)	0.290
Statins, % (n)	59.8 (155)	64.7 (178)	0.245
Diuretics, % (n)	22.8 (59)	15.3 (42)	0.027
Trimetazidine, % (n)	24.3 (63)	27.3 (75)	0.437
Ranolazine, % (n)	17.8 (46)	18.9 (52)	0.732
Nitrate, % (n)	27.8 (72)	16.7 (46)	0.002
Calcium channel blockers, % (n)	18.9 (49)	19.6 (54)	0.834

*ACEI: Angiotensin-converting enzyme inhibitor; ARB: angiotensin receptor blocker.*

**Table 2:** – Comparison of laboratory parameters and lipid indexes of the study groups

Variable	SVG disease (+) (n=259)	SVG disease (-) (n=275)	p
Hba1c, %	7.2 ± 1.9	7.0 ± 2.0	0.296
Fasting glucose level, mg/dL	174 ± 108	164 ± 94	0.249
Hemoglobin, g/dL	13.2 ± 1.64	13.1 ± 1.84	0.205
Hematocrit, %	39.1 ± 4.82	38.6 ± 4.97	0.259
White blood cell count, x10^3^/mL	8.7 ± 2.26	8.5 ± 2.1	0.553
Platelet count, x10^3^/mL	244 ± 68	237 ± 65	0.206
Neutrophil count, x10^3^/mL	5.5 ± 1.78	5.3 ± 1.93	0.307
Lymphocyte count, x10^3^/mL	2.1 ± 0.8	2.1 ± 0.7	0.377
Monocyte count, x10^3^/mL	0.7 ± 0.21	0.7 ± 0.26	0.860
Mean platelet volume, fL	8.8 ± 0.96	9.9 ± 1.12	0.123
Red cell distribution width, fL	15.7 ± 2.71	14.7 ± 2.14	0.182
Total cholesterol, mg/dL, median (25^th^–75^th^ percentile)	201 (174–232)	185.0 (165–222)	0.001
LDL-Cholesterol, mg/dL	137.0 ± 34.7	126.8 ± 37.2	0.001
HDL-Cholesterol, mg/dL, median (25^th^–75^th^ percentile)	37.0 (34.0–43.0)	48.0 (42–54.5)	<0.001
Triglycerides, mg/dL, median (25^th^–75^th^ percentile)	203 (156–273)	143.0 (111–189.5)	<0.001
Urea, mg/dL, median (25^th^–75^th^ percentile)	37.0 (29.0–47.0)	37.0 (29.0–42.0)	0.621
Creatine, mg/dL, median (25^th^–75^th^ percentile)	0.90 (0.70.–1.10)	0.80 (0.70–1.0)	0.253
Albumin, mg/dL	3.8 ± 0.49	3.9 ± 0.38	0.174
Alanine transaminase, U/L	19.1 ± 2.93	19.0 ± 3.17	0.899
Aspartate aminotransferase, U/L	23.2 ± 3.61	24.2 ± 3.94	0.966
Serum uric acid, mg/dl	5.7 ± 1.6	5.4 ± 1.7	0.172
AIP	0.73 ± 0.20	0.50 ± 0.23	<0.001
AC	4.44 ± 1.30	3.08 ± 1.06	<0.001
Non-HDL cholesterol	165.7 ± 42.74	144.6 ± 44.44	<0.001

*AC: Atherogenic coefficient; AIP: Atherogenic index of plasma; HDL: High-density lipoprotein; LDL: Low-density lipoprotein; Non-HDL cholesterol: Non-high-density lipoprotein cholesterol.*

For the prediction of SVG stenosis, the cut-off value of 3.4<AC has 79.15% sensitivity and 66.18% specificity; cut-off value of 0.56<AIP has 83.01% sensitivity and 67.53% specificity; HDL-C<40 mg/dL has 64.9% sensitivity and 84.0% specificity; 121 mg/dL<LDL-C has 78.4% sensitivity and 58.9% specificity; and 141<non-HDL cholesterol has 70.66% sensitivity and 54.55% specificity in the ROC curve analyses (Figure). Paired comparisons of ROC analysis show that, although there were no significant differences between AIP and AC, both AIP and AC were better than HDL-C, LDL-C and non-HDL-C at predicting SVG stenosis.

**Figure 1:** – Receiver operating characteristic (ROC) curves for SVG stenosis.

To determine the independent risk factors for SVG stenosis, univariate and multivariate logistic analysis was performed (Table 3). In the multivariate analysis, history of DM, HT, stroke, HF, number of saphenous grafts, HDL-C, LDL-C and non-HDL-C were found to be independent risk factors for SVG stenosis. Moreover, the atherogenic indexes, AIP and AC were found to be independent predictors of SVG stenosis. Although the triglyceride level was significant in univariate analysis, it did not achieve statistical significance in the multivariate analysis.

## Discussion

In the present study, we found that traditional cardiovascular risk factors such as HT, DM, stroke and heart failure were independent predictors of SVG stenosis. Also, we found that HDL-C, LCL-C, non-HDL-C, the number of SVGs and the atherogenic indexes AIP and AC were independent predictors of SVG stenosis. Moreover, we found that API and AC have better performance to predict SVG stenosis than LDL-C, HDL-C and non-HDL-C.

Saphenous vein graft stenosis is a major issue in the short-term and long-term of CABG patients and is related to major adverse cardiovascular events. It is important to identify patients at high risk of SVG stenosis for prevention and treatment strategies. Many risk factors related to SVG stenosis such as surgery-related factors, smoking, HT, HPL, DM, stroke and others have been determined previously. In their studies, Deppe et al. and Kim et al. have shown that surgery-related factors such as vein acquisition techniques or use of extracorporeal circulation were independent predictors of SVG stenosis.^18,19^ (Descrever sigal)

Smoking is a well-known risk factor for atherosclerosis and coronary artery disease.^20-22^ Sen et al. have shown that smoking is an independent risk factor for SVG disease in their multivariate regression analysis.^20-22^ Similar to previous studies, we have shown that smoking is an important risk factor of SVG stenosis.

DM has been shown to be a major cause of atherosclerosis in many studies.^23^ DM can cause SVG stenosis as a result of vascular endothelial dysfunction caused by immune and non-immune pathophysiological mechanisms.^24^ Koshizaka et al.^25^ examined the relationship between DM and graft failure assessed by one-year angiography and 5-year clinical outcome in CABG patients. In this study, they found that SVG failure in the first year was similar between patients with and without DM. However, death, myocardial infarction or revascularization were found to be significantly higher in DM patients at 5-year clinical outcome.^25^ In our study, history of DM was found to be an independent risk factor for SVG stenosis.

Dyslipidemia is a well-known risk factor for atherosclerosis.^23^ There is increasing evidence that low HDL-C and high LDL-C, total cholesterol and triglycerides play a role in the progression of atherosclerosis and coronary artery disease.^26^ Similarly, low HDL-C with high LDL-C has been shown to be associated with SVG stenosis.^27,28^ Among these parameters, LDL-C level should be recommended to be selected as a treatment target.^26^ However, after reducing LDL-C to the recommended levels, 50% remnant cardiovascular risk remains, thus encouraging researchers to find new arteriosclerosis predictors.^29^ Compared to the single lipid parameters, the comprehensive lipid indexes, such as AIP, AC and non-HDL, are considered to be better predictors of atherosclerosis and coronary artery diseases.^30,31^

In our study, similar to previous studies, low HDL-C and high LDL-C levels were found to be independent risk factors for SVG stenosis. In addition, high level of non-HDL-C, another atherogenic risk factor, was found to be an independent predictor of SVG stenosis. Non-HDL-C is not the first target in lipid-lowering therapy and it may be a secondary target when patients reach recommended LDL-C levels.^26^ In our study, the mean LDL-C level of the patients was found to be above the recommended values for secondary prevention. This shows that patients did not receive the necessary lipid-lowering treatment, and those who received it did not use effective doses for LDL-C, which is the primary target.

As it is known, small density LDL (sdLDL) can accumulate and oxidize more easily on the vessel wall compared to LDL-C. Previous studies have shown that sdLDL is a marker that predicts atherosclerosis and can be used clinically.^26^ However, it is complicated to measure sdLDL in blood and its high cost limits its clinical use. AIP obtained from the TG/HDL-C logarithm strongly reflects the balance between atherogenic and protective lipoproteins. A previous study showed that AIP was inversely related to LDL-C diameter.^32^ For this reason, AIP can be used as an easy indicator in coronary artery disease. AIP can also be routinely calculated from lipid parameters and used as a surrogate of LDL particle size without causing extra costs.

In our study, we found that AIP is an independent indicator of SVG stenosis and had a better performance predicting SVG stenosis than LDL-C and non-HLD-C. Based on these results, we have shown that AIP obtained by a simple calculation can be used to predict SVG stenosis. In previous studies, average AIP values have differed.^15,33-35^ These differences may be due to the non-homogeneity of selected patient groups or drug use. Another reason may be the difference in the burden of atherosclerosis in the population. In our study, we only evaluated patients with CABG under high atherosclerotic risk. As a result, the mean AIP value of our study was higher than that of studies previously published.

Atherogenic Coefficient (AC) is a measure of cholesterol using LDL-C, VLDL-C, IDL-C lipoprotein fractions with respect to HDL-C.^36^

It reflects the atherogenic potential of the entire spectrum of lipoprotein fractions. As emphasized above, non-HDL-c is the second target of therapy after LDL-C as per the ESC (European Society of Cardiology) guidelines, especially in individuals with hypertriglyceridemia.^26^ Hence, this simple index could provide valuable information to identify subjects at risk for cardiovascular diseases. In our study, we found that mean AC was significantly higher in the SVG (+) stenosis group than in the SVG (-) group, and was an independent predictor of stenosis. Also, AC has shown better performance in ROC analysis than non-HDL. This result shows that AC, which is a simple index, should be considered to predict SVG stenosis.

## Limitations

The present study has some limitations. Firstly, this is a retrospective single-center study. However, the number of patients is high, which increases the value of the study. Secondly, we did not compare the results with healthy control subjects, which could show the normal value of AIP and AC in our healthy population. Thirdly, because there were no clinical endpoints in the study, it is not possible to evaluate the impact of AIP and AC for clinical endpoints. Hence, other studies are needed to evaluate the impact of these indexes in CABG patients.

## Conclusion

In the present study, we show that HT, DM, stroke, heart failure and HDL-C, LDL-C and non-HDL-C were independent predictors of SVG stenosis, similar to previous studies. Also, to the best of our knowledge, this is the first study which shows an association between SVG stenosis and the atherogenic indexes AIP and AC. Moreover, we have found that the atherogenic indexes AIP and AC have better performance in predicting SVG stenosis than standard lipid parameters LDL-C, HDL-C, and non-HDL-C.
